# Fractional Brownian motion and multivariate‐t models for longitudinal biomedical data, with application to CD4 counts in HIV‐positive patients

**DOI:** 10.1002/sim.6788

**Published:** 2015-11-10

**Authors:** Oliver T. Stirrup, Abdel G. Babiker, James R. Carpenter, Andrew J. Copas

**Affiliations:** ^1^ MRC Clinical Trials Unit at UCLUniversity College LondonLondonU.K.; ^2^ Department of Medical StatisticsLondon School of Hygiene & Tropical MedicineLondonU.K.

**Keywords:** CD4 counts, HIV, longitudinal data, missing data, random effects models, residuals

## Abstract

Longitudinal data are widely analysed using linear mixed models, with ‘random slopes’ models particularly common. However, when modelling, for example, longitudinal pre‐treatment CD4 cell counts in HIV‐positive patients, the incorporation of non‐stationary stochastic processes such as Brownian motion has been shown to lead to a more biologically plausible model and a substantial improvement in model fit. In this article, we propose two further extensions. Firstly, we propose the addition of a fractional Brownian motion component, and secondly, we generalise the model to follow a multivariate‐t distribution. These extensions are biologically plausible, and each demonstrated substantially improved fit on application to example data from the Concerted Action on SeroConversion to AIDS and Death in Europe study. We also propose novel procedures for residual diagnostic plots that allow such models to be assessed. Cohorts of patients were simulated from the previously reported and newly developed models in order to evaluate differences in predictions made for the timing of treatment initiation under different clinical management strategies. A further simulation study was performed to demonstrate the substantial biases in parameter estimates of the mean slope of CD4 decline with time that can occur when random slopes models are applied in the presence of censoring because of treatment initiation, with the degree of bias found to depend strongly on the treatment initiation rule applied. Our findings indicate that researchers should consider more complex and flexible models for the analysis of longitudinal biomarker data, particularly when there are substantial missing data, and that the parameter estimates from random slopes models must be interpreted with caution. © 2015 The Authors. *Statistics in Medicine* Published by John Wiley & Sons Ltd.

## 1 Introduction

Longitudinal data are commonly analysed using linear mixed models, as formalised by Laird and Ware 1, with ‘random slopes’ models (also including random intercepts) particularly common in the biomedical literature. However, the standard random slopes model makes a strong assumption about the relationship between the outcome variable and time, that is, that this follows a separate linear trajectory for each individual with independent normally distributed errors for each observation point. This underlying assumption is implausible in many biomedical scenarios, and the use of more realistically complex models to account for patterns of variability in the data may allow more information to be gained and lead to a reduction of variance and bias in the estimation of model parameters, particularly in the presence of missing data.

In this paper, we focus on modelling the progression of CD4 cell counts in human immunodeficiency virus (HIV)‐positive patients prior to treatment. These are a type of white blood cell for which counts are monitored over time in order to evaluate the progress of the disease and state of the immune system. Statistical analyses of CD4 cell count data are used to evaluate the natural history of HIV infection and to inform epidemiological simulations. Observational datasets of pre‐treatment CD4 cell counts obtained in clinical practice are usually subject to a high degree of attrition with increasing time from diagnosis, as patients drop out of the cohort because of treatment initiation, loss to follow‐up or death. Furthermore, the timing of observations can be very irregular between and within patients, meaning that flexible statistical structures are required in order to adequately describe patterns of variability in the data.

Taylor *et al.*, 2 proposed the addition of a scaled Brownian motion component to a random slopes linear mixed model, finding that this led to a significant improvement in model fit in terms of Akaike's information criterion for a dataset of 722 measurements obtained from 87 seroconverters, patients who had been observed to transition from an HIV‐negative to HIV‐positive state. Babiker *et al.*, 3 fitted such a model to a dataset of CD4 observations from over 15000 seroconverters and used this to generate CD4 data for simulated cohorts of patients in order to carry out sample size and power calculations for a clinical trial randomising subjects to different treatment initiation rules. Taylor *et al.* also investigated the use of an integrated Ornstein–Uhlenbeck process, of which Brownian motion is a special case, as did Wolbers *et al.*, 5. Fractional Brownian motion is an alternative flexible generalisation of the standard Brownian motion process 6, but its use within the linear mixed model framework has not been investigated. Fractional Brownian motion may be useful for modelling CD4 or other biomarker data as, unlike the integrated Ornstein–Uhlenbeck process, it can allow more erratic variation over time than does simple Brownian motion.

A common finding when assessing the goodness of fit of a statistical model based on the normal distribution, including linear mixed models for the analysis of longitudinal data, is the observation of heavier tails than expected on diagnostic plots of residuals. A natural extension to the standard linear mixed model is to allow the set of observations for each individual as a whole to follow a multivariate‐t distribution. The use of such a model for multivariate regression analysis was proposed by Lange *et al.* and was further developed as an extension of the linear mixed model by Welsh and Richardson 8 and Pinheiro *et al.*. None of these papers included the use of non‐stationary stochastic process components for the modelling of longitudinal biomarker data.

The multivariate‐t distribution was used by Wang and Fan 10 to model CD4 counts in a small sample of 30 HIV‐positive patients taken from a historic trial of antiretroviral (ART) medication. Here, observations were recorded on a regular schedule, and Wang and Fan used a random slopes structure with an additional first‐order autoregression parameter for the residual error. The same authors have also reported the fitting of a similar multivariate‐t model for both CD4 and CD8 cell counts with a second‐order autoregressive structure to a sample of 50 patients from the same historic dataset using a Bayesian approach 11. Matos *et al.* reported the use of a multivariate‐t model for right‐censored HIV RNA assays in untreated patients with acute infection using a nonlinear random effects model for the mean with independent error terms; their model was fitted to 830 observations in 320 individuals. We hypothesised that combining the use of a multivariate‐t model with the addition of a non‐stationary stochastic process component would lead to a further substantial improvement in model fit for pre‐treatment CD4 data. The inclusion of a stochastic process component in the model is important to reflect the erratic trajectories of the CD4 counts of individual patients over time.

Verbeke and Lesaffre found that estimation of fixed effects parameters using linear mixed models is consistent in the presence of non‐normal distributions for the random effects, although they presented a correction to the estimated covariance matrix for the parameter estimates when non‐normality of random effects is suspected 13. Jacqmin‐Gadda *et al.* used simulations to show that inference for fixed effects is robust to misspecification of the error distribution when using linear mixed models in some situations 14. However, these analyses did not take into account the potential for missing or unbalanced data where this is dependent on the observed values of the outcome variable (i.e. data that are ‘missing at random’ (MAR) in Rubin's terminology 15). Gurka *et al.* showed that using overly simplistic covariance structures for linear mixed models can lead to inflation of the Type I error rate even for large samples in the absence of missing data 16. There is therefore a motivation to further investigate biases that may arise from the application of overly simplistic models to realistic datasets that include censoring. This is an important issue for the analysis of observational pre‐treatment CD4 counts in which the timing of censoring from the dataset due to treatment initiation is likely to be strongly linked to the preceding observed values for each individual and the statistical inferences drawn may be more dependent on model choice.

We aimed to further develop the available statistical models for longitudinal biomedical data, incorporating both fractional Brownian motion processes for flexible modelling of intra‐individual variation and multivariate‐t distributions to relax the assumption of multivariate normality. The motivating dataset of pre‐ART CD4 counts used for analysis is introduced in Section 2. Theoretical characteristics of the models fitted and methods for maximum likelihood estimation are described in Section 3. Checking of model adequacy for the data under investigation is crucial, particularly in the presence of missing data. Residual diagnostics for models based on the multivariate‐t distribution are discussed, and novel methods are proposed for the critical evaluation of such models in Section 4. Application of the models developed to the dataset of pre‐ART CD4 counts is described in Section 5, informing simulation studies that are presented to demonstrate differences in predictions made by the more complex models regarding the timing of treatment initiation in population cohorts and to show that the application of simpler models can lead to substantial bias in parameter estimates when there is censoring dependent on observed values of the outcome variable. Practical and methodological implications of the work are discussed in Section 6.

## 2 Dataset

We demonstrate the use of the statistical methods developed through a reanalysis of the dataset of pre‐ART CD4 counts described by Babiker *et al.*, 3, comprising all available measurements prior to the occurrence of acquired immune deficiency syndrome (AIDS)‐defining illness or initiation of ART up to December 2007 from 21 cohorts (originating from 12 countries) participating in the Concerted Action on SeroConversion to AIDS and Death in Europe (CASCADE) study 17. Only patients with a well‐estimated date of HIV seroconversion are included in the CASCADE study, providing a natural ‘zero’ time in each patient for statistical modelling. The total dataset includes 89176 CD4 count observations in 15274 individuals. However, only 3955 (4.4%) measurements from 789 (5.2%) patients were recorded at a time of more than 10 years, and so we chose to model only those CD4 measurements obtained up to 10 years from the time of seroconversion. This resulted in a dataset of 85221 measurements in 15164 individuals. A further 365 observations were excluded for which an identical CD4 measurement was recorded only 1 day after the previous count for that patient, as these were found to cause problems with model estimation and were assumed to result from data‐entry errors, resulting in a dataset of 84856 measurements for analysis.

The CD4 cell counts are measured as cells per microlitre, and we followed established practice in modelling the counts on a square‐root scale 3. As an illustrative example, the CD4 measurements were modelled only in terms of time from seroconversion, expressed as continuous in years, although it would be possible to include other predictive variables. The median number of CD4 observations per individual in the analysed dataset was 4, with a range of 1–57 and an interquartile range of 2–8. There was no rigid pattern to the timing of observations in each patient, with a median interval between measurements of 112 days (interquartile range, 70–182). The highly unbalanced nature of the dataset and the irregular observation schedule necessitate the use of flexible modelling strategies that can accommodate such features. Visual inspection of the CD4 data suggests that the trajectories over time for each individual do not follow predictable paths and that there may be between‐patient differences in variability over time, motivating the combination of stochastic process components and the multivariate‐t distribution, respectively, as presented in this paper. A total of 9831 (64.8%) patients were censored from the dataset at initiation of ART, 1111 (7.3%) because of a recorded AIDS event and 318 (2.1%) at death. Two thousand four hundred and forty‐four (16.1%) patients can be considered lost to follow‐up (with no clinic visit recorded for 12 months and no censoring event), and the remaining 1460 (9.6%) were in follow‐up at the time that the data were gathered.

We hope that the models developed will form the basis for improved epidemiological simulations, as required for the planning of clinical trials and population health analyses, and provide more accurate estimates of the mean CD4 count over time were there to be no censoring of data. Furthermore, the characterisation and quantification of within‐patient and between‐patient variability in CD4 count trajectories may help develop understanding of the natural history of untreated HIV.

## 3 Stochastic process and multivariate‐t models

### 3.1 Characteristics of Brownian motion and related processes

#### 3.1.1 Scaled Brownian motion

In a mathematical sense, Brownian motion (also known as a Wiener process) is a non‐stationary stochastic process that constitutes a continuous‐time generalisation of a simple random walk 18, in which successive increments are independent of the history of the process. When considered in terms of a given set of observation points (these may be irregularly spaced in time), a scaled Brownian motion process, denoted *W*_*t*_ at time *t*, is defined by the following properties:

$$\begin{array}{cc} W_{0} & =0\\ W_{t}-W_{s} & ∼N(0,\kappa (t-s))\text{for}0\leq s<t\text{,}0<\kappa . \end{array}$$

The process starts at zero at time zero (*t* = 0), and increments of the process are stationary, independent (for disjoint periods of time) and normally distributed with mean zero and variance equal to the difference in time between observation points scaled by a positive constant factor *κ*. The following characteristics arise from these conditions:

$$\begin{array}{cc} E[W_{t}] & =0\\ \mathrm{Var}[W_{t}] & =\mathrm{\kappa t}\\ \text{Cov}[W_{s},W_{t}] & =\kappa \times \min(s,t). \end{array}$$

The distribution of a set of *n* observations relating to a given series of time points therefore follows a multivariate normal (MVN) distribution with a mean vector of *n* zeros and covariance matrix defined by the formulae given. As such, Brownian motion is an example of a Gaussian process and can be readily incorporated into the theoretical framework of linear mixed models, as will be discussed in Section 3.2.

#### 3.1.2 Scaled fractional Brownian motion

Fractional Brownian motion represents a generalisation of a Brownian motion process in which increments for disjoint time periods are not constrained to be independent, although they do remain stationary. The process was introduced by Mandelbrot and van Ness^6^. The characteristics of a fractional Brownian motion process are determined by an additional parameter, referred to as *H* or ‘the Hurst index’, that may take a value in the range (0,1). Standard Brownian motion represents a special case of fractional Brownian motion, corresponding to $H=\frac{1}{2}$. As for standard Brownian motion, the expectation of the value of the process is zero for all points in time.

**Figure 1:**
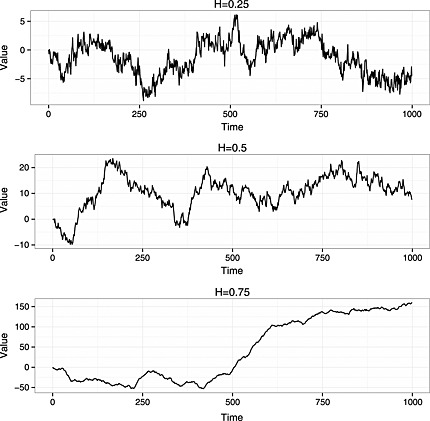
Realisations of fractional Brownian motion processes with varying values of *H* and scale parameter fixed at 1. A finite set of 1000 observations was generated in each case.

When $H<\frac{1}{2}$, successive increments of the process are negatively correlated. This has the consequence, firstly, that the path of the trajectory appears ‘jagged’ and, secondly, that realisations of the process tend to revert towards the mean of zero. For $H>\frac{1}{2}$, successive increments of the process are positively correlated. This means that the path of the process has a relatively ‘smooth’ appearance, and also that individual realisations of the process tend to diverge away from the mean of zero. Illustrative simulated realisations of fractional Brownian motion processes generated with varying values of *H* are shown in Figure 1.

As for Brownian motion, a positive scale parameter (*κ*) can be added to the standard definition of fractional Brownian motion, corresponding to the variance of the process at *t* = 1. We may then characterise the properties of the process as follows:

$$\begin{array}{cc} W_{0} & =0\\ E[W_{t}] & =0\\ \mathrm{Var}[W_{t}] & =\kappa |t|{}^{2H}\\ \text{Cov}[W_{s},W_{t}] & =\frac{\kappa }{2}\left(|s|{}^{2H}+|t|{}^{2H}-\left|t-s\right|{}^{2H}\right). \end{array}$$

Fractional Brownian motion is defined as a continuous‐time stochastic process. However, as we are concerned with modelling biomedical measurements obtained at specific time points, we have focused here on the properties of the process relating to a finite set of observations. As for simple scaled Brownian motion, scaled fractional Brownian motion is a Gaussian process that follows a MVN distribution for any given set of observation points, with expectation zero and covariance matrix as defined.

### 3.2 Marginal distribution for stochastic process models

For models incorporating Gaussian processes such as Brownian motion, the fact that the marginal distribution of the full vector of observations of the outcome variable is MVN means that parameter estimation can be achieved through adjustment of the methods used for standard linear mixed models. The linear mixed model for longitudinal data can be expressed in the following form 1:

(1) $$\begin{array}{cc} y_{i} & =X_{i}\beta +Z_{i}b_{i}+e_{i}\\ b_{i} & ∼\mathrm{MVN}(0,\;\Psi )\\ e_{i} & ∼\mathrm{MVN}(0,\;R_{i}). \end{array}$$

Here, **y**_*i*_ represents the vector of *n*_*i*_ observations for the *i*th individual, **X**_*i*_ represents their design matrix for the ‘fixed effects’ parameters ***β***,**Z**_*i*_ represents the subset of the columns of the design matrix associated with the ‘random effects’ for each individual **b**_*i*_ and **e**_*i*_ is the vector of residual errors for each measurement occasion. The vectors of random effects **b**_1_,**b**_2_,...,**b**_*N*_ and residual errors **e**_1_,**e**_2_,...,**e**_*N*_ for each of the *N* individuals are independent of one another. It can be easily shown that this formulation leads to the following marginal distribution for **y**_*i*_:

$$y_{i}∼\mathrm{MVN}(X_{i}\beta ,\;Z_{i}\Psi Z_{i}^{T}\;+\;R_{i}).$$

When linear mixed models are fitted to longitudinal data, it is common to assume that the residual errors for each observation within each individual, **e**_*i*_, are independent and with constant variance, *σ* ^2^, that is, **R**_*i*_ as defined in (1) is equal to $\sigma^{2}I_{n_{i}}$. However, other forms for **R**_*i*_ are widely used, particularly for the analysis of longitudinal or spatial data. An example is provided by the exponential decay correlation structure 19, for which the elements (*r*_*jk*_) of **R**_*i*_ are calculated as a function of the ‘distance’ *s* between each pair of observations (in the context of longitudinal data this would be the time difference) and a ‘range’ parameter *γ* as follows:

$$\begin{array}{c} r_{\mathrm{jk}}=\sigma^{2}\;\exp\left(-\frac{s_{\mathrm{jk}}}{\gamma }\right). \end{array}$$

Alternatively, the remaining variability in the model, once the random effects have been accounted for, can be subdivided into a component relating to a Gaussian process (independent of other model components) with expectation zero for all time points and an independent residual error for each observation (here assumed to have constant variance); this effectively just creates a class of parameterisations for **R**_*i*_. Defining ***Σ*_*i*_** as the covariance matrix resulting from the chosen Gaussian process and set of time points **t**_*i*_ for the *i*th individual, the linear mixed model can then be expressed as follows:

(2) $$\begin{array}{cc} y_{i} & =X_{i}\beta +Z_{i}b_{i}+W_{i}[t_{i}]+e_{i}\\ b_{i} & ∼\mathrm{MVN}(0,\;\Psi )\\ W_{i}[t_{i}] & ∼\mathrm{MVN}(0,\;\Sigma_{i})\\ e_{i} & ∼\mathrm{MVN}(0,\;\sigma^{2}I_{n_{i}}) \end{array}$$

with marginal distributio

$$\begin{array}{cc} y_{i} & ∼\mathrm{MVN}(X_{i}\beta ,\;Z_{i}\Psi Z_{i}^{T}\;+\;\Sigma_{i}\;+\;\sigma^{2}I_{n_{i}}). \end{array}$$

### 3.3 Multivariate‐t distribution for longitudinal data

There are a number of multivariate generalisations of the univariate‐t distribution, and a thorough review of this topic is provided by Kotz and Nadarajah 20. However, we shall refer to the *multivariate‐t distribution* as that with the probability density function as follows:

$$\begin{array}{c} f\left(y_{i};\mu_{i},V_{i},v\right)=\frac{\Gamma \left(\left(v+n_{i}\right)/2\right)}{\Gamma \left(v/2\right)v^{n_{i}/2}\pi^{n_{i}/2}\left|V_{i}\right|{}^{1/2}\left(1+\frac{1}{v}\left(y_{i}-\mu_{i}\right){}^{T}V_{i}^{-1}\left(y_{i}-\mu_{i}\right)\right){}^{(v+n_{i})/2}}\text{.} \end{array}$$

Where *n*_*i*_ represents the length of the random vector **y**_*i*_( $\in R^{n_{i}}$), **V**_*i*_ is a *n*_*i*_×*n*_*i*_ positive‐definite scale matrix, ***μ***_*i*_ is a *n*_*i*_×1 location vector and *v* is a degrees of freedom parameter. The mean of the distribution is ***μ***_*i*_ if *v* > 1 and otherwise undefined, and the variance of the distribution is $\frac{v}{v-2}V_{i}$ if *v* > 2 and otherwise undefined. This is the most commonly used definition of the multivariate‐t distribution.

In the present context, the mean vector ***μ***_*i*_ will be represented as **X**_*i*_ ***β***, that is, a function of a design matrix **X**_*i*_ and vector of parameters ***β***. As for linear mixed models based on the normal distribution, the scale matrix **V**_*i*_ can be divided into components relating to a random effects structure and a residual error structure, $Z_{i}\Psi Z_{i}^{T}$ and **R**_*i*_, respectively. Pinheiro *et al.* consider the situation in which the degrees of freedom parameter may vary between subgroups of individuals, but we shall assume that this is a single constant 9.

If a vector of observations **y**_*i*_ follows a multivariate‐t distribution

$$y_{i}∼t_{n_{i}}\left(X_{i}\beta ,\;V_{i},\;v\right),$$

then this can alternatively be represented as a hierarchical model in which **y**_*i*_ follows a MVN distribution conditional on a gamma‐distributed variable *τ*_*i*_ (with parameters given for ‘shape’ and ‘rate’, respectively) as follows 9:

(3) $$\begin{array}{cc} y_{i}|\tau_{i} & ∼\mathrm{MVN}\left(X_{i}\beta ,\;\frac{1}{\tau_{i}}V_{i}\right)\\ \tau_{i} & ∼\text{gamma}\left(\frac{v}{2},\;\frac{v}{2}\right). \end{array}$$

In the context of the models proposed, combining variance components related to random effects, stochastic processes and measurement error (i.e. $V_{i}=Z_{i}\Psi Z_{i}^{T}\;+\;\Sigma_{i}\;+\;\sigma^{2}I_{n_{i}}$), this is equivalent to

$$\begin{array}{cc} y_{i} & =X_{i}\beta +Z_{i}b_{i}+W_{i}[t_{i}]+e_{i}\\ b_{i}|\tau_{i} & ∼\mathrm{MVN}\left(0,\;\frac{1}{\tau_{i}}\Psi \right)\\ W_{i}[t_{i}]|\tau_{i} & ∼\mathrm{MVN}\left(0,\;\frac{1}{\tau_{i}}\Sigma_{i}\right)\\ e_{i}|\tau_{i} & ∼\mathrm{MVN}\left(0,\;\frac{1}{\tau_{i}}\sigma^{2}I_{n_{i}}\right)\\ \tau_{i} & ∼\text{gamma}\left(\frac{v}{2},\;\frac{v}{2}\right). \end{array}$$

As noted by Pinheiro *et al.*, 9, it directly follows from the hierarchical form of the model that

(4) $$\begin{array}{ccc} \tau_{i}|y_{i} & ∼\text{gamma}\left(\frac{v+n_{i}}{2},\;\frac{v+\delta_{i}^{2}\left(\theta \right)}{2}\right),\\ \text{where} & \delta_{i}^{2}\left(\theta \right) & =\left(y_{i}-X_{i}\beta \right){}^{T}V_{i}^{-1}\left(y_{i}-X_{i}\beta \right). \end{array}$$

Here, ***θ*** represents the parameter vector that includes ***β*** and determines the construction of **V**_*i*_. From the standard properties of a gamma distribution, it can be seen that

$$E\left(\tau_{i}|y_{i}\right)=\frac{v+n_{i}}{v+\delta_{i}^{2}\left(\theta \right)}.$$

### 3.4 Maximum likelihood estimation and software

As the likelihood function for the multivariate‐normal or multivariate‐t linear mixed‐effects model has a closed form, whatever the structure of **V**_*i*_, it is possible to directly apply Newton–Raphson‐type optimisation procedures. Although finite differencing can be employed, the use of analytically derived exact gradients (with respect to the model parameters) in Newton–Raphson‐type procedures typically greatly improves stability and speed of convergence. However, in some situations, such as incorporating stochastic process components into the multivariate‐t linear mixed effects model, the analytic derivation of the gradients is not trivial. In addition, once an analytic form for each of the gradient terms has been derived, it is required that this be programmed into the computational procedure for the optimisation in an efficient manner.

An alternative method is provided by automatic differentiation, whereby a computer program is structured in such a way that it can automatically calculate the derivatives of a mathematical function to the same degree of accuracy as analytical derivatives (to machine precision) 21. In essence, this is achieved through application of the chain rule to each of the elementary operations that comprise the calculation of the objective function (i.e. the log‐likelihood function). The open‐source Automatic Differentiation Model Builder (ADMB) software (ADMB Foundation, Honolulu, HI, USA) allows optimisation for any statistical model that has a closed form differentiable log‐likelihood function 22 (the software also includes functionality for models without a closed form for the likelihood that is not employed in this paper). For any given model, the user is required to write a ‘template’ file defining a program to calculate the log‐likelihood in terms of the data and the set of unknown parameters to be estimated based on the C++ language; additional statistical and mathematical functions (including matrix and vector functions and operations) are provided by the software to facilitate this. A zip file containing several example template files and a simulated dataset is provided online (Supplementary Data File S1).

For all models presented in Section 5, maximum likelihood estimates of the parameters were obtained using the ADMB software (Version 10.1). The ‘R2admb’ package 23 was used to run analyses and manage results through the R statistical computing environment. Starting values are required for all parameters when using ADMB. These were obtained by using approximate values from a model fit for the initial ‘random slopes’ linear mixed model (including random intercepts) from the *nlme* package for R, and subsequent models were fitted using parameter estimates from the previous simpler model as the initial value. When fitting models with a Brownian motion component, an initial value of 1 was used for the scale parameter, and for models with fractional Brownian motion, an initial value of 0.5 was used for the H index. For models based on the multivariate‐t distribution, an initial value of 10 was used for the degrees of freedom parameter. An R package (covBM) that will allow the implementation of all MVN models described in this paper is under development by the authors.

The ‘fixed effects’ for each model are the intercept (*β*_0_) and a slope (*β*_1_) parameter. For the ‘random effects’ covariance/scale matrix (***Ψ***) for each model, U_00_ and U_11_ represent the variance of the random intercepts and random slopes, respectively, for each individual, with *ρ* representing the correlation between them. For the multivariate‐t models, this interpretation holds conditional on scaling by the vector of unobserved latent variables ***τ***. Models were parameterised using log‐transformations of U_00_ and U_11_ and a generalised logistic transformation of *ρ*. For all models, the residual error term was parameterised using log(*σ*) (i.e. the log of the residual standard deviation). The exponential decay correlation structure was parameterised using the log of the range parameter (*γ*), and Brownian motion models (including fractional) used the log of the scale parameter (*κ*). Fractional Brownian motion was parameterised using the logistic transformation of H. A log transformation was used for the degrees of freedom parameter in multivariate‐t models. For all model parameters, confidence intervals are reported derived from the estimated asymptotic MVN distribution based on the observed information on the transformed scales.

## 4 Residual diagnostics for multivariate‐t models

The evaluation of diagnostic plots of the residuals resulting from fitted statistical models forms an important part of model criticism and development. Such plots can be used to check the adequacy of fitted models to describe the data under investigation and, when problems are observed, to suggest how further improvements might be made. This is particularly important in the present context in which there is interest in understanding patterns of variability within and between individuals as well as ensuring correct inference for fixed effects parameters.

### 4.1 Subject‐level residuals

Much of the focus regarding the use of multivariate‐t linear mixed effects models has been on providing robust inference for the fixed effects; this follows from the fact that individuals with observations that are further from the mean are down‐weighted in the estimation of the fixed effects parameters. Lange *et al.* were concerned with achieving robust multivariate regression and suggested the use of diagnostic residual plots that indicated whether the fitted model adequately reflected the presence of outlying sets of measurements (i.e. corresponding to the various measurements conducted on a single individual) 7. They point out that for a normal linear mixed model, the statistic

$${\hat{\delta }}_{i}^{2}\left(\theta \right)=\left(y_{i}-X_{i}\hat{\beta }\right){}^{T}{\hat{V}}_{i}^{-1}\left(y_{i}-X_{i}\hat{\beta }\right)$$

for each individual would asymptotically follow a χ^2^ distribution with *n*_*i*_ degrees of freedom. However, under a multivariate‐t model, the statistic $\frac{{\hat{\delta }}_{i}^{2}\left(\theta \right)}{n_{i}}$ would asymptotically follow an F‐distribution with *n*_*i*_ and $\hat{v}$ degrees of freedom. Lange *et al.* transform these statistics to standard normal deviates and then use quantile–quantile (Q–Q) plots to assess model fit. A similar approach was used by Wang and Fan 10. Such plots can demonstrate the inadequacy of the normal linear mixed‐effects model to describe the observed data. However, the plots do not directly show whether the multivariate‐t model correctly describes variability between individual measurements.

### 4.2 Measurement‐level residuals

We propose that the gamma–normal formulation of the multivariate‐t model, as given in (3), can be also used to assess whether the multivariate‐t distribution fully describes the patterns of variability observed for all individual measurements in a dataset. As the observations for the *i*th individual are assumed to follow a MVN distribution conditional on *τ*_*i*_, one option is to use empirical Bayes estimates (i.e. the mean of the predicted posterior distribution) of the *τ*_*i*_ as follows:

$${\hat{\tau }}_{i}=\frac{\hat{v}+n_{i}}{\hat{v}+{\hat{\delta }}_{i}^{2}\left(\theta \right)}$$

to estimate the normal covariance matrix ( ${\hat{V}}_{i}^{\prime }$) for each individual

$${\hat{V}}_{i}^{\prime }=\frac{1}{{\hat{\tau }}_{i}}{\hat{V}}_{i}.$$

This could then be used to transform the marginal residuals for the *i*th individual (i.e. $y_{i}-X_{i}\hat{\beta }$) as for a normal linear mixed model using the inverse of a Cholesky decomposition of the covariance matrix (as suggested by Fitzmaurice, Laird and Ware 24), with the transformed residuals for all individuals displayed in a Q–Q plot. However, assuming the empirical Bayes estimates of the *τ*_*i*_ to be correct for all individuals might result in misleading conclusions in a similar manner to that which can be observed when evaluating the empirical Bayes estimates of random effects in a normal linear mixed model (e.g. as reported by Verbeke and Lesaffre 25). An alternative would be to draw a number of repeated samples from the predicted posterior distribution of the full vector of ***τ***, using each sample to generate a full set of ${\hat{V}}_{i}^{\prime }$ matrices and corresponding Cholesky‐transformed marginal residuals. The sets of transformed marginal residuals could then be used individually to generate multiple Q–Q plots or used together to derive a single Q–Q plot showing the distribution of ‘observed quantiles’ over multiple realisations of the ***τ***.

The gamma–normal formulation of the multivariate‐t model provides another route to model checking through the separate evaluation of each individual in the dataset. Assuming that the model parameters are known, then the transformed marginal residuals using the inverse of the Cholesky decomposition of the scale matrix for each individual, **V**_*i*_, are normally and independently distributed with mean **0** and variance $\frac{1}{\tau_{i}}$(conditioned on *τ*_*i*_) as follows:

$$\begin{array}{cc} V_{i} & =L_{i}L_{i}^{T}\\ L_{i}^{-1}\left(y_{i}-X_{i}\beta \right)|\tau_{i} & ∼\mathrm{MVN}\left(0,\;\frac{1}{\tau_{i}}I_{n_{i}}\right). \end{array}$$

Hence, for a model that correctly describes the data, separate Q–Q plots (with respect to the standard normal distribution) of these transformed residuals for each individual should each indicate a normal distribution (with differing variance). For small datasets, it may be possible to create multipanel graphics that simultaneously display the Q–Q plots for all individuals, but for larger datasets, it would be necessary to select a random sample of individuals for inspection. This approach will be more effective when there are a greater number of observations per individual, as it is difficult to assess the assumption of normality for very small samples. This reflects the fact that the presence of a greater number of observations per individual in a dataset will provide more information as to whether there truly is a difference in underlying variability between individuals, as represented by the values of *τ*_*i*_. This technique could also be used for fitted MVN models, using a Cholesky decomposition of the marginal covariance matrix for each individual, in order to assess whether the multivariate‐t distribution might be appropriate for the data.

The assessment of measurement‐level residuals is particularly important when the motivation for an analysis is to be able to make predictions regarding future individual measurements or to simulate datasets in which the exact pattern of values within each individual is critical. The use of subject‐level residuals may be sufficient for multivariate regression analysis (for example, a defined set of different patient characteristics at a single time point in each individual), but for the analysis of longitudinal data we believe that measurement‐level residuals should also be investigated. Examples of the residual plots proposed are presented in Section 5. For these plots, calculations were carried out in R, and graphics were generated using the ggplot2 package for R (Version 0.9.3.1) 26.

## 5 Application and implications of modelling strategy

### 5.1 Set of models fitted

The initial model fitted was a standard linear mixed‐effects model including correlated random intercept and slope terms and independent measurement error terms of constant variance. An exponential delay correlation structure was considered for the error terms of this model, and the initial model was then extended to also include either a scaled Brownian motion process or a scaled fractional Brownian motion process. The equivalent set of four models was then fitted using a marginal multivariate‐t distribution, that is, with the scale matrix **V**_*i*_ structured in the same manner but assuming an unobserved scaling variable for each individual as described in Section 3.3.

### 5.2 Results and diagnostic checks

Table 1 shows the results of linear mixed models (including stochastic process extensions), with marginal MVN distribution, fitted to the pre‐ART CASCADE data. Nested models are compared using the likelihood ratio test; as only a single parameter is being added to the model in each of the comparisons presented, the critical value for change in 2×log‐likelihood (2*Δ* *ℓ*) at the 5% significance level is only 3.84. Non‐nested models are compared using the Bayesian information criterion (BIC) statistic, using the total number of observations in the dataset for the calculation of the penalty term; this is supported by the derivation of Cavanaugh and Neath 27.

**Table 1:** Summaries of extended linear mixed models (with marginal multivariate‐normal distribution) fitted to square‐root transformed pre‐antiretroviral therapy CD4 measurements from the Concerted Action on SeroConversion to AIDS and Death in Europe dataset.

		Random slopes +	Random slopes +	Random slopes +
	Random slopes +	exp. cor. +	Brownian motion+	fBM +
	measurement error	measurement error	measurement error	measurement error
*β*_0_	24.13 (24.02 to 24.24)	24.12 (24.01 to 24.23)	23.81 (23.7 to 23.92)	23.82 (23.71 to 23.92)
*β*_1_	−1.36 (−1.4 to −1.33)	−1.35 (−1.38 to −1.31)	−1.15 (−1.18 to −1.11)	−1.15 (−1.19 to −1.12)
*U*_00_	33.68 (32.65 to 34.73)	33.22 (32.2 to 34.27)	28.69 (27.72 to 29.7)	27.46 (26.46 to 28.51)
*ρ*	−0.39 (−0.41 to −0.36)	−0.38 (−0.41 to −0.35)	−1 (−1 to 1)	−0.59 (−0.63 to −0.54)
*U*_11_	1.62 (1.54 to 1.71)	1.54 (1.46 to 1.62)	0.20 (0.16 to 0.24)	0.58 (0.49 to 0.68)
*σ*	2.76 (2.74 to 2.77)	2.79 (2.77 to 2.81)	2.28 (2.26 to 2.29)	2.01 (1.94 to 2.07)
*γ*	—	0.03 (0.03 to 0.03)	—	—
*κ*	—	—	7.00 (6.78 to 7.22)	9.32 (8.78 to 9.91)
H	—	—	—	0.30 (0.27 to 0.33)
*ℓ*	−232579	−232349	−230109	−230029
BIC	465226	464777	460297	460149

Parameter estimates are given with 95% confidence intervals in parentheses. BIC, Bayesian information criterion; exp. cor., exponential decay correlation structure for residual error term; fBM, fractional Brownian motion; *ℓ*, log‐likelihood.

The addition to the initial random slopes model of an exponential decay correlation structure for the residual variance resulted in a significant improvement in model fit (2*Δ* *ℓ* 460 for 1 degree of freedom (df), *P*<0.001). However, the addition of a Brownian motion component to the random slopes model led to a greater increase in log‐likelihood (2*Δ* *ℓ* 4940 for 1 df, *P*<0.001), with a subsequently lower value of BIC for this model. A further improvement in model fit was observed when the Brownian motion component was generalised to a fractional Brownian motion process (2*Δ* *ℓ* 160 for 1 df, *P*<0.001). As such, the fractional Brownian motion model was found to have the lowest BIC of the fitted linear mixed models. A ‘random slopes + integrated Ornstein–Uhlenbeck process + measurement error’ model was also considered but was found to return the special case of a Brownian motion process (i.e. with a very large estimate for the *α* parameter 2).

**Figure 2:**
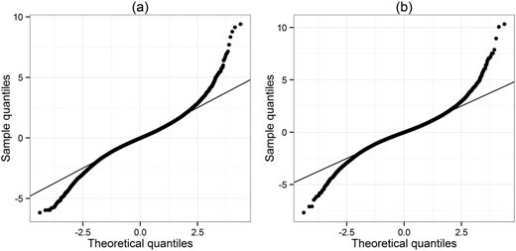
Quantile–quantile plots of Cholesky‐transformed residuals from (a) the ‘random slopes + measurement error’ and (b) the ‘random slopes + fractional Brownian motion + measurement error’ linear mixed model fitted to the pre‐antiretroviral therapy CD4 counts from the Concerted Action on SeroConversion to AIDS and Death in Europe dataset. Plots are generated with respect to a standard normal distribution, and the line of equality is shown.

It is of particular interest that the estimate of the *H* parameter for the model incorporating a fractional Brownian motion process is below 0.5, indicating that successive increments of the process are negatively correlated and hence that the process will tend to revert towards its mean. The mean in this case would include the subject‐specific random effects for the intercept and slope. The correlation between the random intercept and random slope for each individual for the model incorporating a scaled standard Brownian motion process is estimated to be −1.00, which seems rather unnatural. However, when the process is generalised to a fractional Brownian motion, an estimate of ‐0.59 (95% CI, ‐0.63 to ‐0.54) is obtained for this correlation. The Cholesky‐transformed residuals of the commonly used random slopes model and of the best‐fitting linear mixed model, incorporating a fractional Brownian motion component, were analysed to assess the goodness of fit. For both of these models, the Q–Q plot of the Cholesky residuals indicates that their distribution is markedly heavy‐tailed in comparison to the expected standard normal under a correctly specified model (Figure 2).

Summaries of the multivariate‐t distribution models fitted to the pre‐ART CASCADE data are provided in Table 2. As for the MVN models, the fractional Brownian motion model was found to have the lowest BIC of the fitted multivariate‐t distribution models. Furthermore, all of the multivariate‐t models were found to have lower BIC values than all of the normal linear mixed models. The difference in 2*ℓ* between the normal and the multivariate‐t ‘random slopes + fractional Brownian motion + measurement error’ models is 8298, indicating a significant and substantial improvement in model fit (1 df, *P*<0.001). Note that these models can be considered nested as the multivariate‐t model is equivalent to the MVN model as the degrees of freedom parameter tends to (positive) infinity.

**Table 2:** Summaries of multivariate‐t distribution models fitted to square‐root transformed pre‐antiretroviral therapy CD4 measurements from the Concerted Action on SeroConversion to AIDS and Death in Europe dataset.

		Random slopes +	Random slopes +	Random slopes +
	Random slopes +	exp. cor. +	Brownian motion+	fBM +
	measurement error	measurement error	measurement error	measurement error
*β*_0_	23.77 (23.67 to 23.87)	23.76 (23.66 to 23.86)	23.57 (23.47 to 23.67)	23.59 (23.49 to 23.69)
*β*_1_	−1.27 (−1.31 to −1.24)	−1.23 (−1.27 to −1.2)	−1.10 (−1.13 to −1.07)	−1.11 (−1.14 to −1.07)
*U*_00_	23.82 (22.99 to 24.69)	22.83 (22 to 23.68)	20.3 (19.5 to 21.14)	18.82 (17.98 to 19.7)
*ρ*	−0.37 (−0.4 to −0.34)	−0.36 (−0.39 to −0.33)	−1 (−1 to 1)	−0.51 (−0.55 to −0.47)
*U*_11_	1.17 (1.1 to 1.23)	1.01 (0.95 to 1.08)	0.12 (0.1 to 0.15)	0.49 (0.43 to 0.55)
*σ*	2.25 (2.23 to 2.27)	2.32 (2.3 to 2.35)	1.88 (1.86 to 1.9)	1.45 (1.35 to 1.55)
*γ*	—	0.07 (0.06 to 0.07)	—	—
*κ*	—	—	5.17 (4.98 to 5.36)	8.02 (7.44 to 8.64)
H	—	—	—	0.23 (0.21 to 0.26)
df	5.64 (5.4 to 5.88)	5.34 (5.12 to 5.57)	5.83 (5.58 to 6.09)	5.76 (5.52 to 6.02)
*ℓ*	−228221	−227705	−226015	−225880
BIC	456521	455501	452121	451862

Parameter estimates are given with 95% confidence intervals in parentheses. BIC, Bayesian information criterion; df, degrees of freedom parameter; exp. cor., exponential decay correlation structure for residual error term; fBM, fractional Brownian motion; *ℓ*, log‐likelihood.

**Figure 3:**
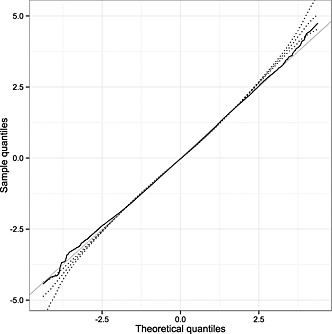
Composite quantile–quantile plot of the distribution of Cholesky‐transformed residuals (for all measurements) from the ‘random slopes + fractional Brownian motion + measurement error’ multivariate‐t distribution model fitted to the pre‐antiretroviral therapy CD4 counts from the Concerted Action on SeroConversion to AIDS and Death in Europe dataset, based on 1000 simulations of the vector of latent variables ***τ***. The dotted lines show the 2.5th, 50th and 97.5th percentiles of the sample quantiles for each theoretical quantile corresponding to the total number of observations; the solid black line shows the sample quantiles derived using the empirical Bayes estimate ( ${\hat{\tau }}_{i}$) for each individual, with the line of equality also displayed in grey.

The estimated degrees of freedom parameter was between 5 and 6 for all of the fitted multivariate‐t models, as expected given the heavy tails observed in the Q–Q plots for the normal linear mixed models. However, the heavy tails could be due to distributional structures other than the multivariate‐t distribution employed, for example the random effects and any Gaussian processes included could follow MVN distributions whilst the residual error terms followed independent t‐distributions. As such, there is a need for further investigation to assess the goodness of fit of the chosen multivariate‐t model with respect to the data. As described in Section 4.2, for the ‘random slopes + fractional Brownian motion + measurement error’ multivariate‐t model, 1000 simulations of the vector of latent variables ***τ*** were generated, based on the predicted posterior distribution in each individual and used to calculate sets of Cholesky‐transformed residuals for the model. The Q–Q plot of the Cholesky residuals derived using the empirical Bayes estimate ( ${\hat{\tau }}_{i}$) for each individual shows a near perfect fit to the standard normal distribution (Figure 3). However, taking quantiles over multiple simulations of ***τ*** indicates the presence of slightly heavier tails than expected.

**Figure 4:**
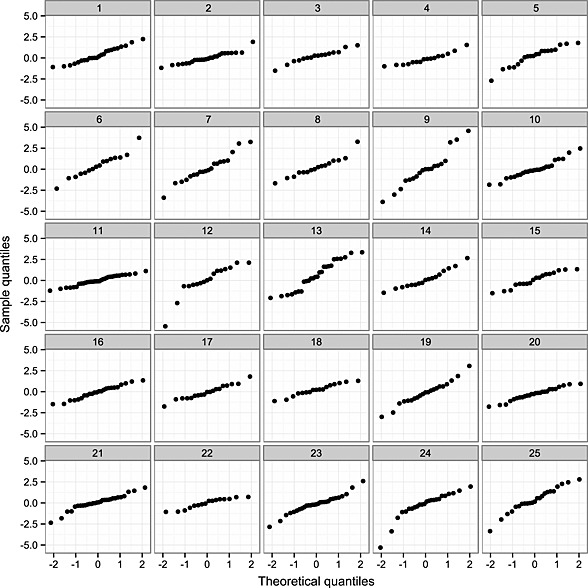
Quantile–quantile plots for the residuals under the ‘random slopes + fractional Brownian motion + measurement error’ multivariate‐t model of 25 randomly selected individuals with greater than 15 observations. The residuals for individual patients have been transformed by the inverse of the Cholesky decomposition of their estimated scale matrix ( ${\hat{\text{V}}}_{i}$) without any correction for the unobserved scale variable *τ*_*i*_. Theoretical quantiles in each case are those from the standard normal distribution.

**Figure 5:**
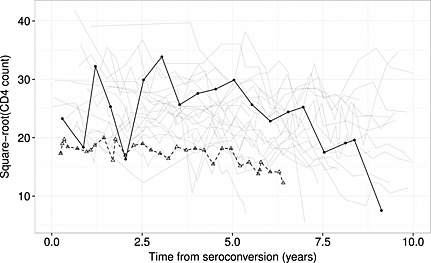
Line plot of the square‐root transformed CD4 counts observed in the random sample of 25 patients with greater than 15 observations (as in Figure 4). The observations for the patients corresponding to Plot 9 (solid black line, filled circles for individual data points) and Plot 11 (dashed black line, open triangles for individual data points) in Figure 4 are made prominent.

The goodness of fit of the ‘random slopes + fractional Brownian motion + measurement error’ multivariate‐t model was further investigated by inspection of Q–Q plots of residuals for individual patients transformed by the inverse of the Cholesky decomposition of their estimated scale matrix ( ${\hat{\text{V}}}_{i}$) without any correction for *τ*_*i*_. As little would be gained by evaluating patients with very few observations, only those with greater than 15 measurements in the dataset were considered; one thousand and forty‐four (6.9%) individuals in the dataset met this criterion. Q–Q plots for 25 randomly selected individuals are shown in Figure 4. Under a correctly specified model, each of the plots should approximately show a straight line of points, with differing slopes between individuals; for the *i*th individual, the expected slope is a function of their unobserved scale variable: $\tau_{i}^{-1/2}$, where $\tau_{i}∼\text{gamma}\left(\frac{v}{2},\;\frac{v}{2}\right)$, with *v* being the degrees of freedom parameter in the multivariate‐t model. These plots suggest that there are indeed differences in overall variability between individuals as implied by the multivariate‐t model; for example, Plot 9 shows a clearly steeper slope than Plot 11. To further illustrate this, the raw data from the 25 sampled patients are shown in Figure 5, with the observations for the patients corresponding to Plots 9 and 11 in Figure 4 made prominent. The ‘Plot 9’ patient has the lowest predicted latent scaling variable ( $\hat{\tau }=0.29$) amongst this subset, corresponding to high variability over time, whilst the ‘Plot 11’ patient has the highest predicted latent scaling variable ( $\hat{\tau }=2.33$) in this group, corresponding to low variability over time.

### 5.3 Simulation study: impact of model choice on treatment initiation predictions

The initiation of ART in HIV‐positive patients is commonly based on the observations of a CD4 count below a given threshold, with the most appropriate cut‐off (or whether treatment should be given immediately upon diagnosis) for any given setting still under debate. As such, there is interest in determining the proportion of patients that will cross any given threshold and initiate ART as a function of time from seroconversion, as this will impact on clinical practice and on the cost of different healthcare strategies. Lodi *et al.*, 28 used random slopes linear mixed models fitted to over 175000 CD4 measurements from the CASCADE cohort (including the data analysed in the present study) to predict the proportion of untreated patients reaching thresholds of <500, <350 and <200 cells/μL with respect to time from seroconversion, reflecting the cut‐offs used in various versions of official guidelines. In this analysis, the distribution of subject‐specific slopes was used to estimate the proportion of patients with ‘true’ CD4 count below each threshold value.

Using their fitted linear mixed model including a Brownian motion component, Babiker *et al.*, 3 investigated the proportion of patients reaching a threshold of <350 cells/μL through simulation of sets of longitudinal measurements for tens of thousands of individuals. This approach has the advantage of allowing realistic assessment of the characteristics of a cohort in practice, and several regimes for the scheduling of measurements and initiation of ART were considered in their simulations. However, the predictions made from the simulations were not directly compared to those that would have been obtained using a normal random slopes model. We have therefore performed a similar analysis based on several of the fitted models in order to investigate this.

Simulated cohorts of individuals were generated based on three MVN models as follows: the random slopes model, the Brownian motion model and the fractional Brownian motion model (with the latter two also including a random slopes structure and all including measurement error). In addition, a cohort was generated using the fitted multivariate‐t fractional Brownian motion model (again, including a random slopes structure and measurement error). For each of these models, data for five million individual patients were simulated based on scheduled measurements being obtained every 4 months for up to 10 years. Data were also generated for measurements 1 month after the scheduled observation in each case for use in the analysis, corresponding to a confirmatory test. CD4 thresholds of <500, <350 and <200 cells/μL for ART initiation were investigated. If a scheduled measurement was observed below a given threshold, then the value 1 month later was assessed to mimic the conduct of an additional confirmatory test as commonly performed in clinical practice. The patient was considered to initiate ART if this second value was also below the threshold.

**Figure 6:**
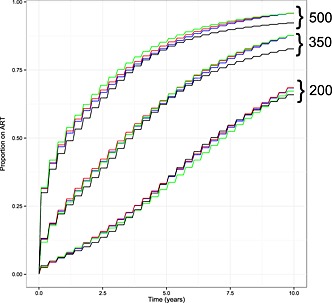
The proportion of HIV‐positive patients predicted to have initiated antiretroviral therapy (ART) as a function of time since seroconversion, based on simulation from the fitted normal random slopes model (black line), Brownian motion model (blue line) and fractional Brownian motion model (red line) and the multivariate‐t fractional Brownian motion model (green line). Results are presented using CD4 thresholds for ART initiation of <500, <350 and <200 cells/μL, as indicated at top right of the graph. Simulations are based on CD4 measurements being obtained every 4 months, with initiation of ART conditional on an additional observation below the cut‐off concerned 1 month after the ‘scheduled’ measurement.

The results of the analysis of the simulated cohorts are presented in Figure 6. The differences in predictions made by each of the fitted models are large enough to have practical implications particularly within a public health or health economics context; for example, using the <500 cells/μL threshold, the proportion of patients on ART 2 years after seroconversion is predicted to be 57% by the normal random slopes model and to be 62% by the multivariate‐t model with fractional Brownian motion. The planning of the Strategic Timing of AntiRetroviral Treatment trial described by Babiker *et al.* made use of predictions of the proportion of patients initiating ART at the 350 cells/μL threshold for which we found only small differences between each of the models that included a stochastic process component (i.e. excluding the standard random slopes model). It is interesting to note that for the 500 and 350 cells/μL cut‐offs, the predictions for the models incorporating stochastic process components converge as time increases towards 10 years, separate to the lower predictions made by the standard random slopes model.

### 5.4 Simulation study: parameter bias in slope estimates

One interesting feature of the various models fitted to the CASCADE pre‐ART CD4 data is that the mean slope (*β*_1_) of CD4 decline is substantially less negative for the linear mixed models that include standard or fractional Brownian motion components (both –1.15) than for the random slopes model (–1.36). The estimated slopes for the equivalent multivariate‐t models were also less steep in each case (Tables 1 and 2). We performed a simulation study to assess the impact of model choice and missing data patterns on this difference, which may indicate apparent bias from the use of simpler models.

It follows from Liang and Zeger 29 that a linear mixed model analysis of longitudinal data will give consistent estimates of the fixed effects given that either there is no missing data or that data is ‘missing completely at random’ (MCAR) (following the terminology of Rubin 15). This also requires the structure of the fixed effects to be correctly specified in the model, but not the exact distribution of observations or covariance between them. Hence, it seems that the substantial differences in slope estimates between different models fitted to pre‐ART CD4 data are due to the presence of missing data for which the missingness is not MCAR, although the framework for missing data terminology is less clear for highly unbalanced datasets without a consistent observation schedule.

It is often postulated that the missingness of observations in pre‐ART datasets can be treated as MAR, that is, that it is independent of the unobserved outcome variable conditional on the observed values of the outcome variable and other covariates included in the model, and that as such the missingness can be ignored under maximum likelihood estimation such as the use of linear mixed models. The MAR assumption is plausible if patients are thought to mainly drop out of the dataset upon initiation of ART, and if this is entirely dependent on their observed CD4 counts. However, the beneficial properties of maximum likelihood‐based inference (i.e. consistency and asymptotic normality and efficiency of estimates) with respect to MAR data are dependent on a correctly specified model for the likelihood. The fact that adding stochastic process components and/or generalising to a multivariate‐t distribution leads to a very substantial improvement in BIC indicates that the standard random slopes model does not correctly describe the covariance structure or probability model for pre‐ART CD4 data.

To further investigate bias in parameter estimates resulting from overly simplistic models, the best‐fitting model (i.e. multivariate‐t with fractional Brownian motion) was assumed to be ‘correct’ and cohorts of patient data simulated from it. CD4 cell count observations were generated from 0 to 5 years, for groups of either 100 or 200 patients and with an annual observation frequency of 1 or 3; five hundred cohorts were generated for each combination. For each simulated cohort, models were first fitted to the complete uncensored data (although this would include impossible negative values), and subsequently to the data following censoring corresponding to ART initiation at CD4 cut‐off values of 200, 350 and 500 cells/μL. The ‘correct’ multivariate‐t model and three normal linear mixed models (the random slopes model, the Brownian motion model and the fractional Brownian motion model) were applied to each simulated cohort under each condition. For the analyses involving censoring, additional confirmatory measurements were generated 1 month after the ‘scheduled’ observations; these were only considered to be observed when the scheduled measurement was below the cut‐off value, and the patient was only censored when the confirmatory value was also below the cut‐off. The censored datasets could therefore be considered to correspond to observations being MAR but not MCAR. As the MAR condition holds for any possible realisation, this scenario meets the ‘everywhere MAR’ definition provided by Seaman *et al.*, 30, allowing valid frequentist likelihood inference. Model fitting was considered to have failed when parameter estimates were not returned or when the covariance matrix of parameter estimates was not positive‐definite.

Limited bias was observed in the estimation of the intercept term when using simplified models and so the results of this analysis are only presented for estimation of the slope parameter *β*_1_. Bias in the estimation of *β*_1_ and the coverage of 95% confidence intervals for this parameter are presented in Table 3. As expected, a lack of bias (or only very minimal bias) and appropriate coverage intervals were observed when the correctly specified model was fitted, even in the presence of censoring. Interestingly, no or only minimal bias was observed when the equivalent normal linear mixed model (including a fractional Brownian motion component) was used. Linear mixed models including a Brownian motion component showed some downward bias in the presence of censoring, with this most marked when censoring was applied using the CD4 cut‐off of 500 cells/μL. Substantial downward biases and poor coverage of confidence intervals were observed when a standard random slopes linear mixed model was applied in the presence of censoring, with the degree of bias clearly linked to the extent of censoring.

**Table 3:** Summary of the results of simulation analyses to assess bias in the estimate of mean slope (*β*_1_) when models that are simpler than the data‐generating process are applied in the presence of ‘missing at random’ censoring.

			RS+ME		RS+BM+ME		RS+fBM+ME		MVT: RS+fBM+ME	
	Prop. cens.	*n* obs.	Failed	*β*_1_ bias	Failed	*β*_1_ bias	Failed	*β*_1_ bias	Failed	*β*_1_ bias
	(median (IQR))	(median (IQR))	(%)	(coverage)	(%)	(coverage)	(%)	(coverage)	(%)	(coverage)
*N*=100, freq=1										
Uncensored	0 (0–0)	600 (600–600)	0	0.013 (93.8)	0.0	0.012 (94.6)	2.6	0.011 (94.0)	1.2	0.012 (96.0)
ART200	21 (18–24)	573 (567–579)	0	−0.100 (88.2)	0.4	−0.021 (96.8)	1.4	0.000 (95.3)	0.2	0.008 (95.4)
ART350	51 (48–55)	505 (493–515)	0	−0.244 (73.0)	1.2	−0.071 (95.1)	2.6	−0.001 (96.5)	1.4	0.000 (95.9)
ART500	77 (75–80)	400 (388–413)	0	−0.384 (72.0)	2.4	−0.146 (94.9)	5.2	−0.022 (94.3)	1.0	−0.019 (93.5)
*N*=100, freq=3										
Uncensored	0 (0–0)	1600 (1600–1600)	0	0.000 (95.4)	0.0	−0.001 (97.0)	4.0	−0.002 (96.0)	2.0	−0.001 (96.7)
ART200	30 (27–34)	1414 (1386–1441)	0	−0.161 (84.4)	0.8	−0.054 (95.8)	4.8	−0.009 (96.0)	3.4	−0.004 (96.1)
ART350	63 (60–66)	1095 (1060–1131)	0	−0.289 (71.8)	0.4	−0.125 (95.0)	7.0	−0.002 (97.8)	2.6	−0.002 (97.9)
ART500	85 (82–87)	724 (687–761)	0	−0.322 (84.6)	2.2	−0.270 (92.0)	9.8	−0.025 (94.5)	3.0	−0.027 (94.2)
*N*=200, freq=1										
Uncensored	0 (0–0)	1200 (1200–1200)	0	0.004 (94.6)	0.0	0.005 (94.4)	1.0	0.004 (93.7)	0.0	0.001 (94.4)
ART200	22 (20–24)	1144 (1136–1152)	0	−0.106 (82.4)	0.2	−0.019 (95.4)	1.2	0.000 (94.1)	0.6	0.000 (95.0)
ART350	52 (49–54)	1008 (993–1023)	0	−0.234 (56.0)	0.4	−0.057 (95.8)	2.4	0.007 (95.3)	0.8	0.005 (95.2)
ART500	78 (76–80)	799 (780–816)	0	−0.360 (56.2)	0.4	−0.118 (92.6)	6.8	0.007 (94.6)	1.6	−0.001 (94.5)
*N*=200, freq=3										
Uncensored	0 (0–0)	3200 (3200–3200)	0	−0.002 (94.6)	0.0	−0.001 (95.6)	2.4	−0.001 (94.7)	0.4	0.000 (92.2)
ART200	30 (28–32)	2840 (2804–2874)	0	−0.161 (66.6)	0.2	−0.054 (93.0)	2.2	−0.012 (94.5)	0.8	−0.005 (92.1)
ART350	62 (60–65)	2197 (2142–2244)	0	−0.300 (44.6)	0.4	−0.127 (88.0)	4.4	−0.014 (96.2)	2.4	−0.007 (96.1)
ART500	84 (83–86)	1454 (1404–1508)	0	−0.337 (70.2)	1.0	−0.243 (86.3)	12.4	−0.004 (93.8)	5.8	−0.017 (93.8)

Bias is calculated as the mean estimate of *β*_1_ minus the true value, and is presented with coverage of nominal 95% confidence intervals in parentheses. For each combination of number of simulated patients (*N*) and annual frequency of observation (freq), 500 cohorts were generated and analysed under different censoring regimes, corresponding to treatment initiation at CD4 cut‐offs of 200 (ART200), 350 (ART350) or 500 (ART500). All cohorts were simulated with a follow‐up of 5 years, including an observation at time zero for each patient. Data were generated according to a multivariate‐t distribution (MVT) incorporating a fractional Brownian motion (fBM) process and measurement error (ME) and, alongside a model of the correct form, normal linear mixed models were fit with a random slopes (RS) structure alone and with RS in combination with Brownian motion (BM) and fBM processes. Model fitting was considered to have failed when parameter estimates were not returned or when the covariance matrix of parameter estimates was not positive‐definite. IQR, interquartile range; *n* obs., total observations included in analysis per simulated cohort; Prop. cens., proportion of patients in simulated cohort subject to censoring before 5years.

A summary of the standard deviations of point estimates for the mean slope and the average estimated standard error for this parameter in the simulations is also provided as supplementary material (Table S1). There were not large discrepancies between these two measures of the standard error. The mean slope estimates from the correctly defined model showed slightly lower variance than the estimates from the incorrectly defined models in any given situation, but the scale of these differences seems relatively small compared with the large biases observed.

The differences in slope estimates observed between models under the censoring conditions in this simulation study correspond to the differences observed between the models when applied to the real dataset. This provides supporting evidence that special attention should be given to the probability model used, and in particular the covariance structure, when analysing a dataset for which there are substantial missing data that are not MCAR. These simulations imply that an analysis using a wrongly specified model might incorrectly indicate differences between two groups in their average rate of decline if they have been subject to different censoring mechanisms. We carried out an additional investigation in which two groups of either 100 or 200 patients each were simulated with three observations per year, with the first group subject to censoring at the ‘200 cut‐off’ whilst the ‘500 cut‐off’ was applied for the second group. Other details of the simulation and model fitting were as previously described, but two additional ‘fixed effects’ parameters were added to the models to allow the mean intercept (*δ*_0_) and slope (*δ*_1_) of the second group to differ from the first group (with the true value of these parameters being zero). These simulations confirmed that bias could occur in the estimation of between‐group differences in slope within a single model (estimated bias for random slopes model with 200 patients per group: –0.163, Table S2).

## 6 Discussion

In this study, we have further developed the statistical modelling of longitudinal biomarker data, through application to pre‐treatment CD4 counts in patients with HIV, in which we have shown that the combination of a fractional Brownian motion component and generalisation of the normal linear mixed model to a multivariate‐t distribution leads to substantial improvements in model fit. This novel combination of model features provides additional information regarding the between‐patient and within‐patient variability in observations over time. Evidence is provided for the appropriateness of using a multivariate‐t distribution in the studied dataset through evaluation of novel diagnostic plots. Furthermore, simulation studies are presented to demonstrate the impact of model choice on cohort‐level predictions and on bias in mean slope estimates when data are MAR.

The presence of non‐stationary stochastic process components in models for longitudinal data implies that the progress of the state of the underlying biological system for each individual does not follow a deterministic relationship with time, but rather follows an unpredictable path. This finding seems intuitive in the context of the extremely complex interactions between viral replication and immune system response that influence the CD4 count series that are observed in HIV‐positive patients. When using a fractional Brownian motion component, the H values obtained were less than 0.5, indicating that the process is erratic but displays some reversion towards an underlying mean. The estimates of the degrees of freedom parameter for the multivariate‐t models of between five and six indicate substantial between‐patient differences in variability over time.

Through simulations based on generating data from the more complex fitted model, it is demonstrated that the use of a normal random slopes model is associated with substantial bias in the estimation of the mean slope parameter in the presence of censoring, with the degree of bias strongly dependent on the choice of censoring regime. This is important, as estimates of this parameter are often used as a proxy for rate of decline in health and compared between groups. As initiation of ART is usually dependent on observed CD4 values, the MAR condition is often invoked to argue that likelihood‐based model estimation will lead to valid inferences, but this only holds conditional on the correct specification of the likelihood model. It can therefore be argued that in this context, greater effort should be made to make use of statistical models that adequately describe the distributional and covariance patterns present in the data.

Diagnostic Q–Q plots of Cholesky‐transformed marginal residuals from MVN models fitted to square‐root CD4 counts show very heavy tails, indicating clear violation of the modelling assumptions. We have demonstrated that the use of a multivariate‐t distribution in combination with a non‐stationary stochastic process component leads to a very substantial improvement in BIC with diagnostic Q–Q plots that only indicate relatively mild violation of the model's assumptions. Such models can be fit efficiently and to large datasets using the open‐source ADMB software 22, with this task made easier by the fact that the log‐likelihood of the multivariate‐t distribution is available in closed form. It would be of interest to investigate whether models comprised of different combinations of multivariate‐t and normal distributions could provide a better fit to the data; such models have been previously discussed by Song *et al.*, 31. For example, it may be considered more biologically plausible to fit a statistical model in which the variability of the stochastic process component differs between individuals (i.e. follows a multivariate‐t distribution) but the random effects and measurement error terms do not (i.e. they follow normal distributions). For such models, the likelihood function is not available in closed form, making the computations required for parameter estimation substantially more complex. The implementation and evaluation of such models will be the topic of further research.

Normal linear mixed models including simple or fractional Brownian motion processes cannot be fitted using standard routines in existing statistical software packages, and this is probably responsible for the fact that they have not been widely adopted in practice (at least in the setting of HIV‐research). However, an R package (covBM) that will allow the implementation of such models is under development by the authors. Most software does not offer any standard function for fitting mixed models based on the multivariate‐t distribution, although an R package ‘tlmec’ does exist for fitting models generalised from a normal model with independent error terms of constant variance 12.

Our research has been focused on CD4 cell counts in HIV‐positive patients, but the modelling framework developed may be of use for the analysis of longitudinal data in other biomedical applications. For example, Diggle *et al.* recently described the use of an extended linear mixed model including another non‐stationary stochastic process, integrated Brownian motion, for the analysis of estimated glomerular filtration rates in patients at risk for renal failure 32. The authors provide plots of ‘Cholesky‐standardised’ residuals produced from the application of the model, which show very heavy tails. The multivariate t‐distribution implies differences in the volatility of observations between patients, which may by useful in planning and interpreting the monitoring of biomarkers in HIV and other disease areas.

Whilst it is arguably impossible to claim that any statistical model exactly represents the data‐generating mechanism under investigation, it seems that both the addition of stochastic process components to the standard linear mixed model and the use of a multivariate‐t distribution can be used to gain a greater understanding of longitudinal biomedical data. Such models provide greater flexibility, but require only a small number of additional parameters and follow a model specification that can be interpreted in terms of the underlying biological process; as such, the potential gains in inference and understanding through their use are likely to greatly outweigh any drawbacks of increased model complexity. There is therefore a motivation to develop more efficient methods of fitting such models and to make these more widely available.

## Supporting information

sup Info Item

sup Info Item

sup Info Item
