# Supplementary material for: Fractional Brownian motion and multivariate‐t models for longitudinal biomedical data, with application to CD4 counts in HIV‐positive patients
Source: Stat Med. 2015 Nov 10;35(9):1514–32. doi: 10.1002/sim.6788 (PMC4982109; doi:10.1002/sim.6788)
Supplement: Supplementary file 1 — sup Info Item [file SIM-35-1514-s001.pdf]

Supplementary Table S1 Summary of the SD of slope estimates obtained ($SD(\hat{\beta}_1)$) and mean of standard error estimates ($mean(\widehat{SE_{\beta_1}})$) from the simulation analyses to assess bias in the estimate of mean slope (β_1) when models that are simpler than the data-generating process are applied in the presence of 'missing at random' censoring. For each combination of number of simulated patients (N) and annual frequency of observation (freq), 500 cohorts were generated and analysed under different censoring regimes, corresponding to treatment initiation at CD4 cut-offs of 200 (ART200), 350 (ART350) or 500 (ART500). All cohorts were simulated with a follow-up of 5 years, including an observation at time zero for each patient. Data were generated according to a multivariate-t distribution (MVT) incorporating random slopes (RS), a fractional Brownian motion (fBM) process and measurement error (ME) and, alongside a model of the correct form, normal linear mixed models were fit with a RS structure alone and with RS in combination with Brownian motion (BM) and fBM processes (all with ME).

	RS+ME		RS+BM+ME		RS+fBM+ME		MVT: RS+fBM+ME	
	$SD(\hat{\beta}_1)$	$mean(\widehat{SE_{\beta_1}})$	$SD(\hat{\beta}_1)$	$mean(\widehat{SE_{\beta_1}})$	$SD(\hat{\beta}_1)$	$mean(\widehat{SE_{\beta_1}})$	$SD(\hat{\beta}_1)$	$mean(\widehat{SE_{\beta_1}})$
N=100, freq=1								
Uncensored	0.131	0.131	0.131	0.132	0.131	0.130	0.110	0.115
ART200	0.147	0.146	0.137	0.144	0.135	0.139	0.116	0.122
ART350	0.201	0.184	0.177	0.188	0.179	0.181	0.156	0.158
ART500	0.347	0.292	0.297	0.317	0.311	0.301	0.273	0.268
N=100, freq=3								
Uncensored	0.133	0.132	0.129	0.138	0.130	0.129	0.107	0.110
ART200	0.165	0.153	0.140	0.153	0.137	0.143	0.118	0.121
ART350	0.205	0.203	0.187	0.213	0.178	0.200	0.154	0.168
ART500	0.383	0.340	0.372	0.391	0.363	0.353	0.310	0.304
N=200, freq=1								
Uncensored	0.096	0.093	0.095	0.093	0.094	0.092	0.083	0.081
ART200	0.111	0.103	0.099	0.101	0.100	0.099	0.088	0.086
ART350	0.142	0.130	0.121	0.131	0.126	0.127	0.113	0.111
ART500	0.228	0.207	0.213	0.220	0.211	0.212	0.189	0.189
N=200, freq=3								
Uncensored	0.094	0.093	0.095	0.097	0.091	0.092	0.078	0.077
ART200	0.106	0.103	0.101	0.106	0.094	0.099	0.084	0.084
ART350	0.145	0.130	0.135	0.146	0.131	0.127	0.110	0.118
ART500	0.270	0.207	0.263	0.268	0.268	0.212	0.213	0.215
