# Supplementary material for: Fractional Brownian motion and multivariate‐t models for longitudinal biomedical data, with application to CD4 counts in HIV‐positive patients
Source: Stat Med. 2015 Nov 10;35(9):1514–32. doi: 10.1002/sim.6788 (PMC4982109; doi:10.1002/sim.6788)
Supplement: Supplementary file 2 — sup Info Item [file SIM-35-1514-s002.pdf]

Supplementary Table S2 Summary of simulation study to assess bias in the estimation of between-group differences in intercept (δ_0) and slope (δ_1) within a single model when different patterns of censoring are applied to the two groups. Using the probability distribution of the 'RS+fBM+ME multivariate-t model' fitted to the real dataset, two groups of either 100 or 200 patients each were simulated with three observations per year up to 5 years, with the first group subject to censoring at the '200 cut-off' while the '500 cut-off' was applied for the second group (with corresponding 'confirmatory' observations in each case). Alongside a model of the correct form, normal linear mixed models were fit with a random slopes (RS) structure alone and with RS in combination with Brownian motion (BM) and fractional Brownian motion (fBM) processes (all with ME). 500 iterations of the simulation were performed for each group size. The true value of both δ_0 and δ_1 was set to zero, and were estimated as the difference in mean intercept and slope parameter for the second group relative to the first.

	RS+ME	RS+BM+ME	RS+fBM+ME	MVT:RS+fBM+ME
Group size=100 each				
Bias: $\hat{\delta_0}$	0.091	0.027	0.022	0.020
$SD(\hat{\delta_0})$	0.806	0.771	0.758	0.651
$Mean(\widehat{SE_{\delta_0}})$	0.805	0.765	0.754	0.655
Coverage: δ_0	95.0	94.8	94.5	94.6
Bias: $\hat{\delta_1}$	-0.143	-0.108	0.004	0.004
$SD(\hat{\delta_1})$	0.275	0.270	0.271	0.230
$Mean(\widehat{SE_{\delta_1}})$	0.278	0.294	0.268	0.237
Coverage: δ_1	92.0	95.6	94.5	95.6
Failed (%)	0.0	0.6	2.2	3.8
Group size=200 each				
Bias: $\hat{\delta_0}$	0.04	-0.020	-0.014	-0.007
$SD(\hat{\delta_0})$	0.576	0.554	0.547	0.471
$Mean(\widehat{SE_{\delta_0}})$	0.572	0.544	0.536	0.465
Coverage: δ_0	95.0	95.0	94.6	94.6
Bias: $\hat{\delta_1}$	-0.163	-0.128	-0.016	-0.013
$SD(\hat{\delta_1})$	0.197	0.192	0.189	0.164
$Mean(\widehat{SE_{\delta_1}})$	0.197	0.208	0.190	0.168
Coverage: δ_1	87.0	92.4	95.0	94.8
Failed (%)	0.0	0.0	0.8	0.2

ME, measurement error; MVT, multivariate-t distribution.
